# Pyocyanase

**Published:** 1910-09-15

**Authors:** Carl Ruttloff

**Affiliations:** The Zahntechnische Reform


### PYOCYANASE.

BY DR. CARL RUTTLOFF, FROM THE ZAHNTECHNISCHE REFORM.

TRANSLATED BY CARL E. KLOTZ, D.D.S.,
ST. CATHARINES, ONTARIO.

A new preparation has been placed on the market by the Serum Works and Institute of Bacterio Therapeutics, of Dresden, Saxon, Germany, under the name of Pyocyanase, which appears to fill a long felt want in the Dental Materia Medica.

The effective treatment of Alveolaris Pyorrhea, Stomatitis Gingivitis and kindred diseases of the mouth, have presented many difficulties to the dental practitioner, before which he stood helpless with the usual therapeutical remedies.

Pyocyanase appears to be a remedy that not only simplifies the therapeutic treatment, but promises an effective cure. According to "Emmerich and Loew," Pyocyanase is obtained from a liquid culture of Bacillus Pyocanes several weeks old, through germ separating, filteration and further, through a condensing in vacuum and clarifying. It appears as a dark colored greenish fluorescent liquid, with a pretty high specific gravity and a peculiar jasmine like odor.

Its strong bactericidal power is evident, that notwithstanding a culture of several million cubic centimeters, pneumococcus are completely destroyed in three minutes. Diptheria-bacillus and streptococcus are completely destroyed in ten minutes. Gonococcus, are completely destroyed in five minutes. Vibrio-cholerae, destroyed in five minutes. Dysentery-bacillus, destroyed in three hours. Staphylococcus and Typhoid-bacillus, destroyed in twenty-four hours. While a diluted solution of 1-40 checks markedly, the development of staphylococcus and 1-125 that of diptheria-bacilla, notwithstanding the strong disinfectant power of pyocyanase, it is perfectly harmless, and for that reason, very adaptable for use in the oral cavity.

This new remedy has been used very successfully in the treatment of diptheria, to support the serum in case of extended membrane formation, in grippe epidemics, cerebro spinal meningitis, scarlet fever, etc.

The reports of the gratifying success of pyocyanase led Dr. Reich to experiment with it in dentistry, and his results presented to a convention of dentists, held in Brandenburg, induced me to try the preparation in my own practice, and can state that in the treatment of different cases of pyorrhea and stomatitis, it has never failed me. After two or three applications a decided improvement could be noticed, and from reports received from patients whom I had treated with it prove it to have effected a permanent cure.

In March, 1909, a lady about 42 years of age came to my office with a serious case of pyorrhea in the six lower anterior teeth. Her own dentist having told her that it would be useless to attempt a cure, and nothing could be done but to extract the teeth, but to this she objected. This was in 1907, two years before she came to my office. When I examined her mouth I found a typical case of chronic alveolar pyorrhea. The teeth were very loose, percussion painful, the gums highly inflamed, a quantity of calcareous deposit on the teeth, and on pressure a copius flow of pus. After the fifth treatment there was a great improvement, the teeth became firmer and no discharge of pus. The improvement has continued and the teeth are now almost as firm as ever.

Also in cases of stomatitis, mercurialis, pyocyanase has done good service, that I now use it with patients as a prophylactic, that are sent to me by physicians, prior to an inunction treatment. I also had good results in a case of lead poisoning, when the case has extended to alveolar necrosis of the left lower cuspid, lateral and central. In this case also the discharge of pus and the pain ceased after a few treatments and the teeth were saved.

Before giving my mode of treatment with pyocyanase, I will cite one more case, which is of particular interest and stated by Dr. Reich in his publication, "The Nature of Pyocyanase and the use of it in different forms of Gingivitis and Stomatitis," and which may be considered as difficult a case to treat as ever came into the hands of a dentist.

The patient, a bookbinder by trade, 42 years of age, with whom I was personally acquainted, as he had been one of my patients some time ago, and had treated him, but without success. In the years 1904 to 1906, his gums were loosened and bled at the slightest touch, the teeth were also loose and covered with a slimy and smeary deposit. Physicians whom he had consulted were not of the same opinion in their diagnosis. As the patient had contracted lues in his youth, he was treated with an anti lues treatment, while others diagnosed it a case of metal poisoning. He was treated alternately with chromic acid and with mouth washes, baths and juniper berries, still the disease got worse from day to day. Finally he was sent to Dr. Reich for dental treatment, who found his mouth in a very deplorable condition. The gums were of a deep red color and spongy and covered with a thick purluent fibruous exudation, the interdental gingiva could be separated quite a distance from the teeth, shreds of necrotic fibruous tissue peeled off from the mucous membrane of the cheek and palate, and in some places the bone was exposed. The tongue showed deep purulent fissures. The molars made deep impressions in the cheek, which caused ulceration, and the lip also has several ulcers. The teeth on the right side particularly were so loose that they could almost be moved with the fingers. In the morning the mouth was fairly pasted together by the exudated pus, which had hardened to a crust at the corners of the mouth. The faetor ex ore was unbearable.

After the first pyocyanase treatment there was a decided improvement and in several months later a complete cure was affected, with the exception of several teeth that were too far gone when treatment began. The rest are all firm again. The mucous membrane and tongue have their normal appearance and his general health has improved.

In this case the pyocyanase therapy has done wonders, but on account of the gravity of the disease it required a number of months of anxious treatment to effect a cure.

It certainly must not be expected that every case of alveolar pyorrhea or stomatitis can be immediately relieved unless the whole of the oral cavity is given a thorough examination and put in a proper condition. This brings me to the point of explaining my mode of treatment.

The first step is to thoroughly cleanse the oral cavity and remove every trace of deposit from the teeth, without this, no subsequent treatment is of any use.

After thoroughly disinfecting the mouth with washing and rinsing with peroxide of hydrogen, the teeth to be treated are again washed with a 10 per cent. solution of peroxide of hydrogen and kept dry with rolls of absorbent cotton and lintine. Dr. Reich recommended at this stage to drop a 10 per cent. soluton of peroxide of hydrogen into the pockets with a drop pincette and work it down with a small but blunt instrument and dry with absolute alcohol. Now, place with a drop pincette the concentrated pyocyanase into the pockets with a suitable instrument, work it down to the bottom and leave it there for several minutes without the saliva coming in contact with it, and not to rinse the mouth immediately after the application, so as not to dilute the drug, as it is very much more effective when used in full strength. Patients will not complain about the taste or odor of it. This treatment is repeated when necessary.

From the foregoing, it may be seen that the pyocyanase therapy is a valuable factor in the treatment of all the diseases of the oral cavity. Besides, the splendid bacterecidal property of this remedy it is of great value and importance to the dental profession, on account of it being perfectly harmless and is nonescharotic.—*Dominion Dental Journal.*

